# Whole genome sequence and diversity in multigene families of *Babesia ovis*

**DOI:** 10.3389/fcimb.2023.1194608

**Published:** 2023-08-01

**Authors:** Junya Yamagishi, Onur Ceylan, Xuenan Xuan, Ferda Sevinc

**Affiliations:** ^1^ International Institute for Zoonosis Control, Hokkaido University, Sapporo, Japan; ^2^ Global Station for Zoonosis Control, GI-CoRE, Hokkaido University, Sapporo, Japan; ^3^ Department of Parasitology, Faculty of Veterinary Medicine, University of Selcuk, Konya, Türkiye; ^4^ National Research Center for Protozoan Diseases, Obihiro University of Agriculture and Veterinary Medicine, Obihiro, Japan

**Keywords:** ovine babesiosis, *Babesia ovis*, comparative genomics, multigene families, apicomplexa

## Abstract

Ovine babesiosis, caused by *Babesia ovis*, is an acute, lethal, and endemic disease worldwide and causes a huge economic loss to animal industry. Pathogen genome sequences can be utilized for selecting diagnostic markers, drug targets, and antigens for vaccine development; however, those for *B. ovis* have not been available so far. In this study, we obtained a draft genome sequence for *B. ovis* isolated from an infected sheep in Turkey. The genome size was 7.81 Mbp with 3,419 protein-coding genes. It consisted of 41 contigs, and the N_50_ was 526 Kbp. There were 259 orthologs identified among eight *Babesia* spp., *Plasmodium falciparum*, and *Toxoplasma gondii*. A phylogeny was estimated on the basis of the orthologs, which showed *B. ovis* to be closest to *B. bovis.* There were 43 *ves* genes identified using hmm model as well. They formed a discriminating cluster to other *ves* multigene family of *Babesia* spp. but showed certain similarities to those of *B. bovis*, *B. caballi*, and *Babesia* sp. Xinjiang, which is consistent with the phylogeny. Comparative genomics among *B. ovis* and *B. bovis* elucidated uniquely evolved genes in these species, which may account for the adaptation.

## Background

*Babesia ovis* is a tick-borne intraerythrocytic apicomplexan parasite. It is one of the causative agents of ovine babesiosis in small ruminants. The disease is a worldwide epidemic, especially in Europe, Africa, and Asia. The manifestation is characterized by fever, hemolytic anemia, hemoglobinuria, and icterus (Yeruham et al., 1998; Ahmed et al., 2006; Fakhar et al., 2012; Ranjbar-Bahadori et al., 2012; Sevinc et al., 2013; Fanelli, 2021). Interestingly, it is highly virulent in sheep but milder in goats (Yeruham et al., 1998). However, correlation and etiology among geographic distribution, phenotype, and genotype still need to be elucidated owing to the paucity of genome information. Current countermeasures rely on pharmacotherapy of the affected animals and vector control by acaricides, which poses risks of resistance in both pathogen and vector. Therefore, effective vaccines are also desired but have yet to be available so far. Attenuation by successive blood passages in splenectomized lambs was conducted but was not successful (Sevinc et al., 2014). Latent infection and recurrence after splenectomy were reported (Habela et al., 1990). These observations might provide clues to the difficulty in vaccine development. Potential vaccine antigens designated as *B. ovis*–secreted antigen 1 and *B. ovis*–secreted antigen 2 have been nominated by immune screening (Sevinc et al., 2015a; Sevinc et al., 2015b). Genetic diversity among the species is mainly examined using *18S rRNA* genes, and the resolution is not high enough to reflect the geographic diversity (Tumwebaze et al., 2020; Galon et al., 2022a; Galon et al., 2022b). Whole genome sequence for *B. ovis* is promising in enhancing vaccine development and optimizing genotyping markers as well.

Whole genome sequence is one of the fundamental information in biology, and the genomes of following *Babesia* spp. are publicly available: *B. bigemina*, *B. bovis*, *B. caballi*, *B. divergens B. microti*, *B. ovata*, and *Babesia* sp. Xinjiang (Brayton et al., 2007; Cornillot et al., 2012; Ma et al., 2013; Jackson et al., 2014; Guan et al., 2016; Yamagishi et al., 2017). In terms of disease control, they contribute to the development of diagnostic methods and vaccines, screening of drug targets, and identification of drug-resistant genes. They are also indispensable to understand the evolution and adaptation of the parasites. By comparative genomics among *Babesia* spp., it is demonstrated that there is an expansion of *ves* genes. In *B. bovis*, it has *ves1α* and *ves1β* gene that code Variant Erythrocyte Surface Antigen 1a (VESA1a) and 1b (VESA1b) (Allred, 2019), respectively. According to the genome analysis, there are more than 104 *ves* genes including other groups of *ves*, *ves1γ*, and *ves2* (Brayton et al., 2007). VESA1a and VESA1b are expressed as a set and form a heterodimer that is involved in cell adhesion and pathogenicity (Allred et al., 1993; O’Connor and Allred, 2000; Pedroni et al., 2013). In other *Babesia* spp., they also have species specific *ves* multigene families, namely, *ves1a*, *ves1b*, and *ves2* of *B. bigemina* (Jackson et al., 2014) and *B. ovata* (Yamagishi et al., 2017), *ves1* of *B. divergens* (Jackson et al., 2014) and *ves1* of *Babesia* sp. Xinjiang (Guan et al., 2016), although their function is not well demonstrated (O’Connor and Allred, 2000; Pedroni et al., 2013). In contrast, it is known that there are species-specific gene expansions such as *smorf*, *mtm* type A and B in *B. bovis*, and *mfs in B. bigemina* and *B. ovata* (Brayton et al., 2007; Yamagishi et al., 2017; Hakimi et al., 2020; Hakimi et al., 2022). The *smorf* genes form a multigene family of *B. bovis* that codes small open reading frame protein and locate proximal to the *ves1α* and *ves1β* (Brayton et al., 2007). Their function is not clear (Ferreri et al., 2012), but the involvement in the transcriptional regulation of the *ves* genes is suggested (Allred, 2019). The *mtm* type A and B genes of *B. bovis* are group of genes with eight or more predicted multi-transmembrane domains (Hakimi et al., 2020). They are exported to the surface of infected red blood cells (RBCs) (Hakimi et al., 2022). One of the *mtm* type B is proven to confer drug resistance, and others are speculated to be involved in nutrition uptake (Hakimi et al., 2020; Hakimi et al., 2022). The *mfs* genes code Major Facilitator Superfamily proteins with eight or more predicted multi-transmembrane domains. They widely exist in *Babesia* spp., but the expansion is only found in *B. bigemina* and *B. ovata* (Yamagishi et al., 2017; Hakimi et al., 2020). Their function has not yet been elucidated.

In this study, we assembled a whole genome sequence of *B. ovis* obtained from infected sheep in the field. Comparative genomics among *B. ovis* and related *Babesia* spp. revealed a diversity of multiplied genes that may be involved in host switch and adaptation through the evolution.

## Materials and methods

### Genomic DNA extraction, library construction, and sequencing

*Babesia ovis*–infected blood sample was obtained from the natural case (50% parasitemia levels) located in an area in the Central Anatolian Region of Turkey in which ovine babesiosis is enzootic in 2015. The parasite was monitored by a microscopic examination of Giemsa-stained thin blood smears and verified by the PCR analysis. *B. ovis*–infected blood was washed five times with phosphate-buffered saline (PBS) by centrifugation at 2,000 rpm for 15 min to separate RBCs from other cells, and, then, infected RBCs were diluted 10 times with 0.1% saponin (in PBS) to lyse. It was kept at room temperature for 5 min and centrifuged at 4,000 rpm for 20 min to collect merozoites. The merozoite pellet was diluted with PBS, and DNA extraction was performed by using Phenol Chloroform Isoamyl Alcohol (Sigma P2069) (Green and Sambrook, 2012). MinION libraries were constructed with the Ligation Sequencing Kit 1D, LSK108 (Oxford Nanopore Technologies), and then sequenced with three FLO-MIN106 flow cells. A PacBio library was sequenced using the PacBio RS II sequencer with two SMRT cells. An Illumina library was constructed with a TruSeq DNA PCR-Free kit (Illumina), and 300-bp paired-end sequencing was performed using a MiSeq sequencer (Illumina). Reads with low quality were removed using Trimmomatic v0.36 based on the following parameters: LEADING:20 TRAILING:20 SLIDINGWINDOW:4:15 MINLEN:36.

### RNAseq analysis

Infected blood from the same sheep was dissolved into a PAXgene blood RNA tube, and RNA was purified using the PAXgene Blood RNA Kit (QIAGEN). The quality and quantity of the purified RNA were validated with Bioanalyzer (Agilent). Library construction for RNAseq was performed as per the instruction manual with the TruSeq Stranded mRNA LT Sample Prep Kit (Illumina), and 300-bp paired-end sequencing was performed using a MiSeq sequencer (Illumina).

### *De novo* genome assembly

Obtained fast5 data were base-called to fastq using poretools 0.6.0 (Loman and Quinlan, 2014) or albacore.1.2.6. The fastq derived from MinION and Pacbio preassembled reads and Illumina paired-end reads were assembled using SPAdes-3.11.1 with -k 127 option (Bankevich et al., 2012). The resulting scaffolds were examined to subtract possible host contamination and artifacts based on the following criteria. First, scaffolds with equal or more than 1,000 bp were selected. Second, redundancy among the scaffolds was excluded if more than 90% of the length of each scaffold had more than 80% identity against the other scaffolds. The identity was evaluated by BLASTN (Altschul et al., 1990). Third, scaffolds were excluded if the region showing the highest identity against *B. bovis* or *B. bigemina* was less than 10% of each scaffold. The identity was evaluated by BLASTX (Altschul et al., 1990) using *B. bovis* (Brayton et al., 2007), *B. bigemina* (Jackson et al., 2014), and *Ovis aries* (Archibald et al., 2010) coding sequences and non-redundant protein database provided by National Center for Biotechnology Information (NCBI). Finally, sequencing depth was calculated using BamDeal (https://github.com/BGI-shenzhen/BamDeal) with aligned reads made by bowtie2 version 2.3.4.1 (Langmead and Salzberg, 2012) for the Illumina reads and BWA version 0.7.17 (Li and Durbin, 2010) for the nanopore and PacBio reads, and, then, scaffolds with too low (less than 10) or too high (more than 100) coverage were excluded using the Illumina reads. Scaffolds corresponding to both apicoplast and mitochondria genomes were additionally specified on the basis of sequence similarity. The qualified scaffolds were polished using the Illumina reads with Pilon with 15 iterations until a plateau was reached (Walker et al., 2014). To eliminate redundancy, further quality filtering was performed using Redundans (Pryszcz and Gabaldón, 2016). The qualified contigs were validated using BlobTools v1.1.1 (Laetsch and Blaxter, 2017). The translated amino acids sequences of each contigs were examined using Diamond version 2.1.6 (Buchfink et al., 2021) against NCBI non-redundant protein (nr) database and then subjected to BlobTools as inputs. Integrity of the assembly was validated using BUSCO using the apicomplexa_odb10 dataset (Manni et al., 2021).

### Gene model estimation and functional annotation

The gene model was estimated by AUGUSTUS version 3.1.0 (Stanke et al., 2008). Trained parameters for AUGUSTUS were acquired by webAugustus (Hoff and Stanke, 2013) using the *B. bovis* gene model obtained from PiroplasmaDB version 5.1 (Ma et al., 2013). The RNAseq data were mapped onto the *B. ovis*–assembled draft genome with Tophat2 (Trapnell et al., 2009). The first round of AUGUSTUS was performed to obtain an exon–exon junction database. The second round of AUGUSTUS was performed using the database to obtain the final gene model. Our previous report describes the procedure in detail (Yamagishi et al., 2017). Functional annotation of *B. ovata* genes, including gene ontology (GO) terms, was conducted by Blast2GO (Conesa et al., 2005). tRNA and rRNA genes in the genome were predicted by tRNAscan (Lowe and Eddy, 1997) and RNAmmer 1.2 web servers (Lagesen et al., 2007) with default parameters, respectively. Gene model in the apicoplast and mitochondria genomes was estimated by MiGAP considering their bacterial origin.

### Acquisition of publicly available sequences and gene annotation

For the comparative genomic analysis among apicomplexan parasites, we used genome assembly and annotation released in PiroplasmaDB-37 for *B. bovis* T2Bo strain, PiroplasmaDB-36 for *B. bigemina* BOND strain, PiroplasmaDB-40 for *B. ovata* Miyake strain, PiroplasmaDB-61 for *B. caballi* USDA-D6B2 strain, PiroplasmaDB-40 for *B. microti* RI strain, PiroplasmaDB-46 for *B. divergens* 1802A strain, PiroplasmaDB-55 for *Babesia* sp. Xinjiang, ToxoDB-40 for the *T. gondii* ME49 strain, and PlasmoDB-56 for the *P. falciparum* 3D7 strain.

### Phylogenetic analyses

Orthologous genes conserved among these parasites were specified using OMA version 2.5.0 with the default parameters (Altenhoff et al., 2015). The orthologs coded in the *B. ovis* genome and all the nine parasites above were selected. Amino acid sequences of the orthologs were aligned using MAFFT with the default parameters (Katoh et al., 2002). The resulting gaps were trimmed, and the remaining sequences were concatenated. A phylogenetic tree by the maximum likelihood method with 100 times of bootstrap replications was constructed using MEGA version 10.0.5 (Kumar et al., 2018)

### Synteny analyses

Synteny between *B. ovis* and *B. bovis* genomes were examined using MCScanX (Wang et al., 2012). The identity between the amino acid sequences of the genes was calculated using BLASTP, and gene pairs with e-values less than 1e-100 were selected and then the synteny was visualized by AccuSyn (https://accusyn.usask.ca/). Structural diversity among apicoplast genomes of *B. ovis* (BaOVIS_AP_20230606), *B. bovis* (NC_011395.1), and *Babesia* sp. Xinjiang (KX881914.1) was examined by dotplot using YASS (Noe and Kucherov, 2005). Structural diversity among mitochondrial genomes of *B. ovis* (BaOVIS_MT_20230606), *B. bovis* (EU075182.1), and *Babesia* sp. Xinjiang (KX698108.1) was examined in the same way.

### Uniquely gained and lost genes in *B. ovis*

The uniquely gained genes were defined as the *B. ovis* genes that have no orthologs in either species, as shown below. The first set was closely related to *B. bovis* and *Babesia* sp. Xinjiang. The other set was wider range of *Babesia* spp., *B. bovis*, *B. bigemina*, *B. ovata*, *B. caballi*, *B. divergens*, and *Babesia* sp. Xinjiang. The uniquely lost genes were defined as the genes conserved in *Babesia* spp. mentioned above but lack in *B. ovis.* Those in *B. bovis* were retrieved as representatives. GO enrichment analysis for both gained and lost genes was performed using agriGO (Tian et al., 2017).

### Identification of multigene families

The *ves* and *smorf* multigene families in the *B. ovis* genome were searched using hidden Markov model. The training dataset was constructed on the basis of the public genomes and annotations mentioned above. In detail, *ves1α*, *ves1β*, and *smorf* in *B. bovis* were retrieved from the gff by searching “variant erythrocyte surface antigen-1, alpha,” “variant erythrocyte surface antigen-1, beta,” and “*smorf*,” respectively. *Babesia divergens ves1* genes were retrieved from the gff by searching “variant erythrocyte surface antigen.” *Babesia bigemina ves1a*, *ves1b*, *ves1ba*, and *ves2* were retrieved from the website by Sanger institute (https://www.sanger.ac.uk/resources/downloads/protozoa/babesia-bigemina.html). *Babesia* sp. Xinjiang *ves* was obtained from GenBank sequence MBFZ00000000.1 (Guan et al., 2016). Hidden Markov models for each gene set were constructed, and, then, *ves*-related genes in *B. ovis* were identified using HMMER version 3.3.2 (Mistry et al., 2013). The genes in *B. ovis* and the abovementioned *ves* and *smorf* genes in *B. bovis*, *B. divergens*, *B. bigemina*, and *Babesia* sp. Xinjiang were aligned using BLASTP (Altschul et al., 1990). Those with more than 200-bit score were selected, and mutual similarity was visualized with Gephi using a Fruchterman–Reingold layout (Bastian et al., 2009).

The *mtm* multigene family in *B. ovis* was examined using an algorithm predicting transmembrane domain, TMHMM version 2.0c (Krogh et al., 2001), if there were eight or more predicted transmembrane domains and at least one TM domain average per 100 amino acids. Those in *B. caballi*, *B. divergens*, and *Babesia* sp. Xinjiang identified with the same method. Those in *B. bovis* and *B. bigemina* were retrieved from the previous study (Hakimi et al., 2020). Mutual similarity among them was examined and visualized as mentioned above.

To find other multigene families, the genes in *B. ovis* were aligned to themselves using BLASTP. Those with more than 200-bit score were selected, and mutual similarity was visualized with Gephi using a Fruchterman–Reingold layout (Bastian et al., 2009). Clusters were assigned by Gephi function, and their functions were estimated using BLASTP with nr database.

## Results

### *De novo* assembly of *B. ovis* genome

Using MiSeq, we obtained 1.9 million paired-end reads consisting of 1.1 Gbp. In parallel, a total of 196,077 reads and 295,660 subreads, consisting of 0.6 and 1.8 Gbp, were obtained using MinION FLO-MIN106 and PacBio RS II, respectively.

Using SPAdes assembler, these reads were assembled into 2,920 scaffolds comprising approximately 9.2 Mbp. Following subtraction based on redundancy, possible host contamination, and coverage, 80 scaffolds were qualified. Sequentially, we conducted error correction using the Illumina reads with Pilon. In 15 iterations, the modification reached a plateau resulting in the correction of substitution of 353 nucleotides, 11 insertions, and six deletions, respectively. To eliminate redundancy, further quality filtering was performed using Redundans (Pryszcz and Gabaldón, 2016), and 39 redundant contigs were removed. Coverage, guanine-cytosine (GC) contents, and coding sequences of the qualified contigs were validated using BlobTools (Laetsch and Blaxter, 2017) and confirmed that the coverage and GC contents were consistent except for apicoplast and mitochondria contigs. In addition, the majority of the amino acid sequences encoded by each contig were related to Apicomplexan parasite genes (**Figure S1**), suggesting that there was no migration of sequences other than *B. ovis* in the qualified contigs. The sequencing depth was 27.1, 9.3, and 1.9 for the Illumina, Nanopore and PacBio reads, respectively. Finally, the draft genome consisting of 7,811,629 bp was successfully obtained and designated as Selcuk assembly (**Table 1**). The longest scaffolds, N_50_, and L_50_ were 1,533,616 bp, 525,571 bp, and the fifth longest contig out of the 41 scaffolds, respectively. The integrity of the assembly was validated by BUSCO, and percentages of completeness, fragmented, and missing core genes were 96.6%, 0.9%, and 2.5%, respectively.

**Table 1:** Comparative analysis of genome and gene among representative *Babesia* spp.

	*B. ovis*	*B. caballi*	*B. bigemina*	*B. ovata*	*B. bovis*	*B. sp. Xinjiang*	*B. divergens*	*B. microti*
Genome size (bp)	7,811,629	12,816,698	13,840,936	14,453,397	8,179,706	8,373,550	8,915,963	6,392,438
–Apicoplast genome size (bp)	35,596	31,649	NA	30,355	33,351	30,729	NA	28,657
–Mitochondria genome size (bp)	6,015	5,978	8,037	6,198	6,005	6,020	5,811	11,149
N50 (bp)	525,571	3,243,686	2,541,256	2,090,503	1,797,577	533,301	1,092,625	1,766,409
# of contigs/scaffolds	41	9	483	91	14	215	82	6
# of coding genes	3,419	5,910	5,079	5,031	3,706	3,066	4,129	3,494
# of tRNA	64	65	46	64	69	40	NA	44
# of *5.8S rRNA*	5	9	6	6	9	NA	NA	2
# of *18S rRNA*	1	4	3	3	3	NA	NA	2
# of 28S rRNA	0	3	3	4	3	NA	NA	2
Reference	This study	Under review	Jackson et al., 2014	Yamagishi et al., 2017	Brayton et al., 2007	Guan et al., 2016	Jackson et al., 2014	Cornillot et al., 2012

NA, not available.

### Gene model estimation and functional annotation

In this study, we applied AUGUSTUS, which provides a more reliable gene model estimation supported by experimentally obtained transcriptome (Stanke et al., 2008). A total of 3.8-Mbp paired-end reads were obtained by RNAseq. Of these, 10.7% were successfully mapped to the assembled draft genome using Tophat2. Considering that the blood specimen included host sheep cells together with *B. ovis*–infected RBC, the mapped ratio was reasonable. Two scaffolds had high sequence similarity with significant e-value (“0.0” for both) against the apicoplast and mitochondria genomes of *B. bovis*, respectively, and, then, MiGAP was utilized for gene modeling, considering that they evolved from bacterial symbionts. Collectively, 3,419 coding regions (CDS) were predicted (**Table 1**). It corresponded to the number of CDS in *B. bovis*. tRNAs, *5.8s rRNA*, and *18S rRNA* were estimated in 64, five, and one locus, respectively. Functional annotation of the CDS was performed with Blast2GO. On the basis of the estimation, 2,772 CDS were functionally annotated, whereas the remaining 647 CDS were then simply assigned as hypothetical proteins.

### Phylogenetic and synteny analyses

**Figure 1:**
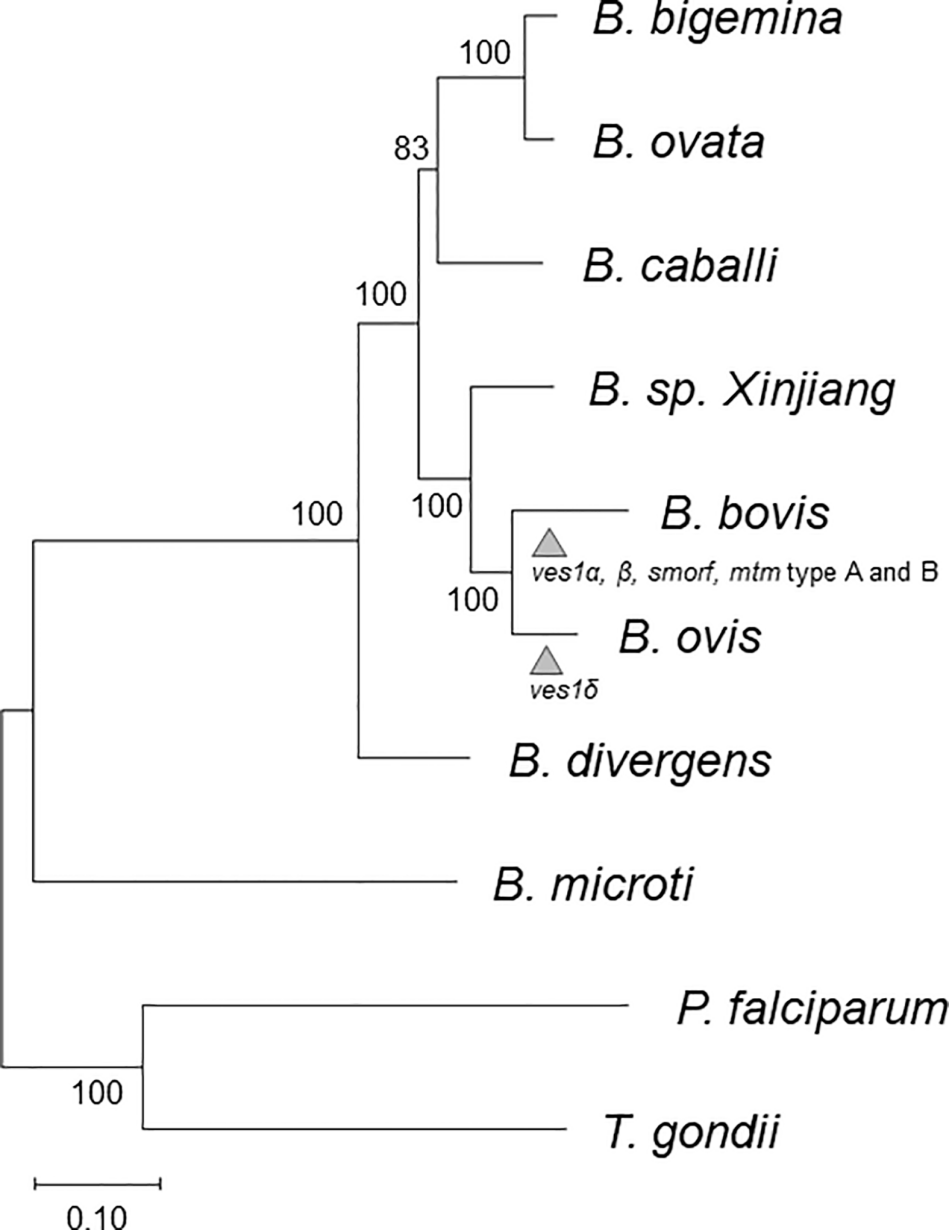
Phylogenetic tree based on 259 orthologs among *B. caballi*, *B. bovis*, *B. bigemina*, *B. ovata*, *B. microti*, *B. divergens*, *B.* sp. Xinjiang, *P. falciparum*, *T. gondii*, and *B. ovis.* The arrowheads represent estimated points for expansion of each gene family. The numbers at the branches represent bootstrap values.

To create an orthology-based phylogenetic tree, we identified 259 single-copy orthologous genes among *B. bovis*, *B. bigemina*, *B. caballi*, *B. microti*, *B. divergens*, *B. ovata*, *Babesia* sp. Xinjiang, *P. falciparum*, *T. gondii*, and *B. ovis* using OMA. Their amino acid sequences were aligned; then, gaps were removed and concatenated; and a phylogenetic tree was constructed (**Figure 1**). This genome-wide analysis revealed that *B. ovis* was phylogenetically most close to *B. bovis.*

**Figure 2:**
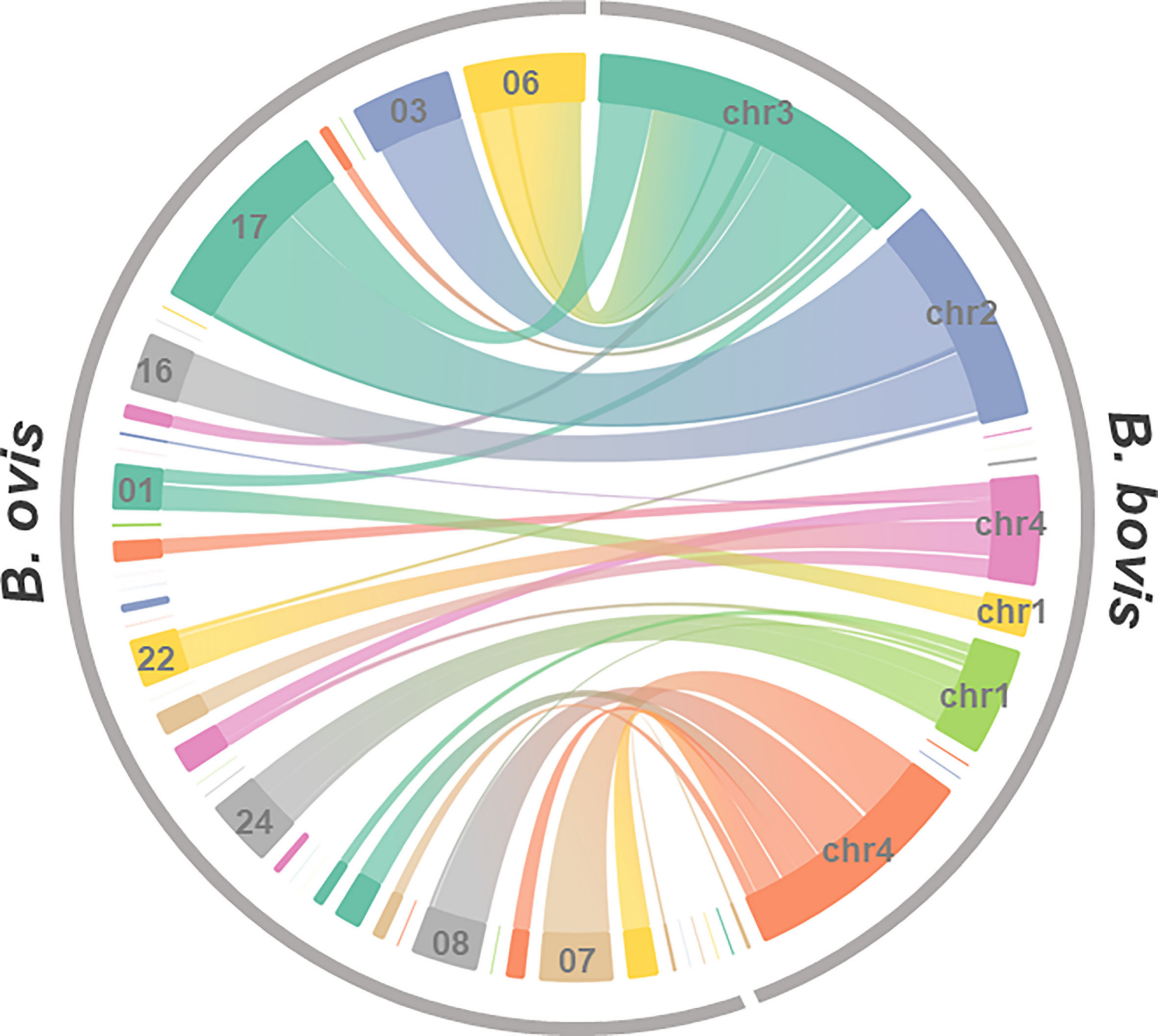
Genome synteny between *B. ovis* and *B. ovata*. Synteny blocks identified by amino acid sequence similarity of genes of both species were linked.

We then examined synteny between *B. ovis* and the closest *Babesia* species, *B. bovis* (**Figure 2**). It implied that synteny blocks among them were generally conserved although better genome assembly is needed to conclude this. In addition, the contig 17 of the *B. ovis* genome diverges from chromosomes 2 and 3 of the *B. bovis* genome, indicating the presence of structural recombination. The synteny of apicoplast and mitochondrial genome among *B. ovis*, *B. bovis*, and *Babesia* sp. Xinjiang was examined as well. Dotplot analyses were applicable due to high sequence identity among them, and their synteny was well conserved (**Figures S2**, **S3**).

### Uniquely gained and lost genes in *B. ovis*

We have identified 281 uniquely gained genes compared to closely related *Babesia* spp., *B. bovis*, and *Babesia* sp. Xinjiang. To evaluate systematic gain in function, we analyzed the 281 genes using gene ontology; however, there was no enrichment compared to the whole ontology in *B. ovis*. We also identified 19 uniquely gained genes in comparison to wider *Babesia* spp. However, there was no specific enrichment as well. We have identified 229 and 57 uniquely lost genes against a close and wide range of *Babesia* spp., but there was not any enrichment as well.

### Multiple-gene families

**Figure 3:**
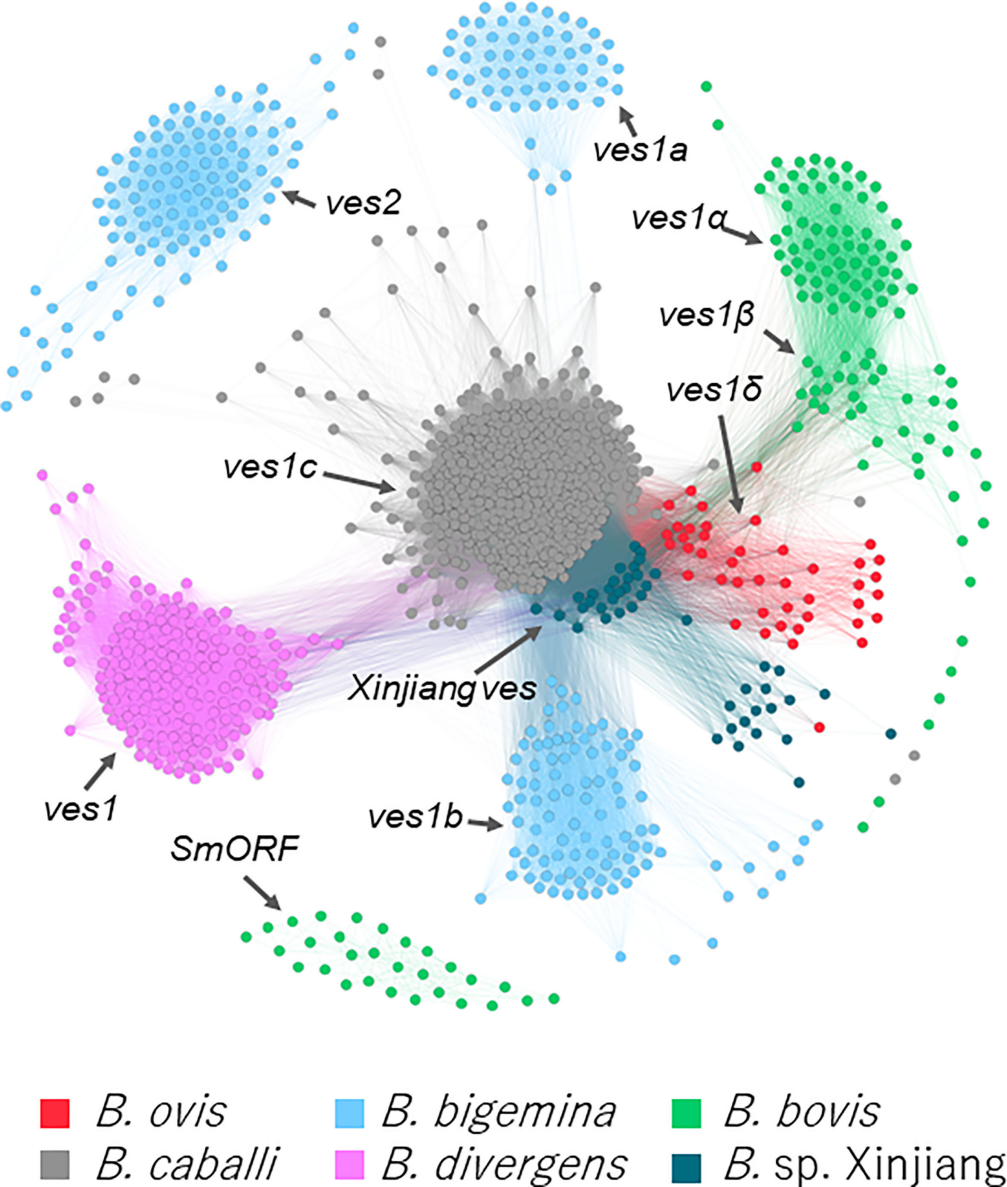
Clustering based on sequence of *ves* -like genes and *smorf* in *B. bigemina*, *B. caballi*, *B. divergens*, *B. bovis*, *Babesia* sp. Xinjiang, and *B. ovis.* Each node represents a protein-coding gene in the five parasites. Edges represent the similarity between connected nodes.

Using hmm model, 43 *ves1*-like genes were identified in *B. ovis* (**Table S1**). Mutual homologies among the *B. ovis ves1*-like genes, *smorf*, and *ves* of *B. bovis*, *B. bigemina*, *B. caballi*, *B. divergens*, and *Babesia* sp. Xinjiang were visualized. It demonstrated that the *B. ovis ves1*-like genes were differentiated from *ves1α* and *ves1β* of *B. bovis* (**Figure 3**). A part of *Babesia* sp. Xinjiang *ves* and *B. ovis ves1*-like genes had similarity; however, they clustered separately from each other.

Genes that encode proteins with multi-transmembrane domains were examined, and 60 genes with eight or more predicted transmembrane domains were identified (**Figure S4**). It is known that *B. bovis* and *B. bigemina* have unique duplicated genes with the multi-transmembrane domains, *mtm* and *msf*, respectively. However, *B. ovis* did not have such duplicated genes.

Other multigene families in *B. ovis* were examined using a similarity search among *B. ovis* genes (**Figure S5**). The biggest cluster was cluster 1, consisting of 16 genes annotated as DEAD box ATP-dependent RNA helicase, followed by clusters 2 and 3 consisting of 10 and 8 genes, respectively. They were annotated as 26S protease regulatory subunit 8 and T-complex protein 1, respectively.

Regarding the expanded *B. ovis ves1*-like genes, those number could be under estimated because the redundancy of the assembled contigs was enough removed. On the other hand, it is also less likely that the expansion of the *mtm* family genes had been overlooked at all. Because the expanded *mtm* genes of *B. bovis* and *B. bigemina* are scattered over the contigs regardless of the size of contig, however, there is no expansion of *mtm* in the *B. ovis* genome. It is true that gene expansion and contraction can only be fully concluded by the chromosome level of the genome; however, it is possible to qualitatively assert the presence or absence of expansions.

## Discussion

Hybrid scheme using long reads and short reads is the current standard for *de novo* genome assembly (Segerman, 2020). We obtained approximately 7.81-Mbp genome sequence consisting of 41 contigs using the approach. The BUSCO analysis revealed that it contained almost all genes in the contigs. However, the number of contigs was much larger than the known chromosome number in *Babesia* spp., which ranges from four or five (Ray et al., 1992; Jones et al., 1997; Depoix et al., 2002; Cornillot et al., 2013). The similar fragmented assembly is reported in *B. ovata* (**Table 1**). In contrast, the hybrid assembly in *B. caballi* gives almost complete assembly. One of the potential factors discriminating the results among them is with or without the sub-cloning step. Through long-term *in vitro* culture, quasi-species can be derived from recombination among chromosomes and accumulated, which can hamper assembly (unpublished data). Indeed, we obtained better assemblies by sub-cloning in *B. bovis* (data not shown). In *B. ovis*, the source of genome DNA was obtained from neither isolated clone nor sub-clone but from wildly infected sheep; therefore, heterogeneity in the genome DNA might be retained in the population.

The *B. ovis* genome sequence coded 3,419 protein-coding genes (**Table 1**). Previous studies using *18S rRNA* genes suggest that *B. ovis* is the closest to *B. bovis*, followed by *Babesia* sp. Xinjiang, although bootstrap values are not definitive (Oosthuizen et al., 2008). This was consistent with our phylogenetic tree described on the basis of 259 homologs among Apicomplexan parasites and demonstrated more precisely (**Figure 1**). The number of genes in *B. ovis* was comparable to those in *B. bovis* and *Babesia* sp. Xinjiang as well. There were some gained and lost genes compared to *B. bovis* and *Babesia* sp. Xinjiang; however, there was no ontology terms that were found to be enriched in this set of genes. These lines of evidence suggest that fundamental biological flamework is conserved among them.

It is known that every *Babesia* sp. except *B. microti* has *ves* multigene family (Brayton et al., 2007; Cornillot et al., 2012; Jackson et al., 2014; Guan et al., 2016; Yamagishi et al., 2017). In *B. ovis*, we identified 43 *ves1*-like genes using hmm model (**Figure 3**, **Table S1**). They made a cluster that was discriminated from other species specific *ves* clusters; therefore, it should be appropriate to name them *ves1δ*. Without *ves1δ* of the *B. ovis* genome, *ves1α* and *ves1β* of *B. bovis* are almost separated from other clusters; however, *ves1δ* filled the gap, suggesting that they evolved continuously, shared the same ancestral genes, and differentiated along with the speciation (**Figure 3**). Similarly, *smorf* and *mtm* A type and B type of *B. bovis* seem to appear after the speciation between *B. ovis* and *B. bovis*. It is speculated that *smorf* is involved in transcriptional regulation for *ves1α* and *ves1β* (Allred, 2019). The fact that *smorf*, *ves1α*, and *ves1β* emerge simultaneously in *B. bovis* but absent in *B. ovis* supported the hypothesis. Other well-described multigene families in *B. bovis* are *mtm* A type and B type (**Figure S4**). It is known that one of the *mtm* is involved in drug resistance, and it is speculated that *mtm* family genes are involved in nutrition uptake (Hakimi et al., 2020; Hakimi et al., 2022). It is interesting to estimate the origin of *smorf* and *mtm* family genes; however, their homologs are not identified among any *Babesia* spp., including the most closely related *B. ovis* (**Figures 3**, **S4**). It is possible to speculate that they were acquired by horizontal gene transfer or that their evolution was too fast to trace back to their ancestral genes. However, more pieces of evidence are required to clarify the role of these factors. On the basis of the comparative genomics among *B. ovis* and related *Babesia* spp., the uniqueness of *B. bovis* was uncovered. Considering that both *B. ovis* and *Babesia* sp. Xinjiang infect the sheep *Ovis*, it is possible to speculate that adaptation from *Ovis* to *Bos* required differentiation of *ves1α* and *ves1β* from ancestors of *ves1δ* and expansion of *smorf* and *mtm* (**Figure 1**). Regarding the expanded *B. ovis ves1δ*, those number could be underestimated because the redundancy of the assembled contigs was enough removed. On the other hand, it is also less likely that the expansion of the *mtm* family genes had been overlooked at all. Because the expanded *mtm* genes of *B. bovis* and *B. bigemina* are scattered over the contigs regardless of the size of contig, however, there is no expansion of *mtm* in the *B. ovis* genome. It is true that gene expansion and contraction can only be fully concluded by the chromosome level of the genome; however, it is possible to qualitatively assert the presence or absence of expansions.

Whole genome sequencing of pathogens provides much more data than conventional single-gene sequencing, which makes significant contributions to the advances in the diagnosis and prevention of diseases (Akoniyon et al., 2022). Pathogen genome sequences provide invaluable and large-scale data for the selection of new diagnostic and drug targets and the identification of antigens to be used in vaccine development studies. Today, although genome sequence information is available for many *Babesia* species including *B. microti*, *B. bigemina*, *B. bovis*, *B. divergens*, *B. ovata*, *Babesia* sp. Xinjiang, and *B. caballi*, this information is not yet available for *B. ovis*. This study introduces the *B. ovis* whole genome sequence, which is 7.81 Mbp in size and contains 3,419 protein-coding genes. In the future, it is strongly anticipated that these data will contribute to increasing novel vaccine and drug targets in the prevention of *B. ovis*–induced babesiosis.

## Data availability statement

The genome sequence and annotation presented in the study are deposited in the NCBI GenBank, accession number BLIY00000000 (https://www.ncbi.nlm.nih.gov/nuccore/BLIY00000000). Corresponding BioProject and Biosample ID are PRJDB7132 (https://www.ncbi.nlm.nih.gov/bioproject/PRJDB7132) and SAMD00128968 (https://www.ncbi.nlm.nih.gov/biosample/SAMD00128968), respectively. Illumina, Nanopore and PacBioreads are available from SRA under the following accession numbers, DRR489302-DRR489304 (https://ddbj.nig.ac.jp/resource/sra-run/DRR489302- https://ddbj.nig.ac.jp/resource/sra-run/DRR489304), respectively.

## Ethics statement

The animal study was reviewed and approved by the Ethics Committee of Selcuk University Faculty of Veterinary Medicine. The samples were obtained as part of the diagnosis of livestock and the informed consent was obtained orally. The infected blood samples were collected in the project supported by the Scientific and Technological Research Council of Turkey (Project No. 113O336). Animal experiments performed following the rules of the Ethics Committee of Selcuk University Faculty of Veterinary Medicine (No. 2013/003).

## Author contributions

JY, XX, and FS conceived and designed the study. OC and FS conducted sample collection. JY, OC, and FS performed the experiments. JY, OC, XX, and FS conducted the literature search, performed data extraction and analysis, and interpreted the results. JY drafted and wrote the manuscript. OC, XX, and FS critically reviewed the manuscript for important intellectual content and revised the manuscript. All authors contributed to the article and approved the submitted version.

## Supplementary material

Supplementary Figure 1 Profile of the 41 assembled contigs of B. ovis by BlobTools. It was confirmed that no contigs were found to have low sequence depth or deviate from the average GC% except apicoplast and mitochondrial genome.

Supplementary Figure 2 Dotplot among apicoplast genomes of *B. ovis. B. bovis* and *Babesia.* sp. Xinjiang.

Supplementary Figure 3 Dotplot among mitochondrial genomes of *B. ovis. B. bovis* and *Babesia.* sp. Xinjiang.

Supplementary Figure 4 Clustering based on sequence of genes with more than eight multi-transmembrane domains in *B. bigemina*, *B. caballi*, *B. divergens*, *B. bovis*, *Babesia* sp. Xinjiang, and *B. ovis.* Each node and edge represent a protein-coding gene and similarity between connected nodes, respectively.

Supplementary Figure 5 Clustering based on sequence in *B. ovis* genes. Each node and edge represent a protein-coding gene and similarity between connected nodes, respectively. The clusters were colored if they consisted of five or more genes.
