# Supplementary figures and images for: Whole genome sequence and diversity in multigene families of Babesia ovis
Source: Front Cell Infect Microbiol. 2023 Aug 1;13:1194608. doi: 10.3389/fcimb.2023.1194608 (PMC10471129; doi:10.3389/fcimb.2023.1194608)

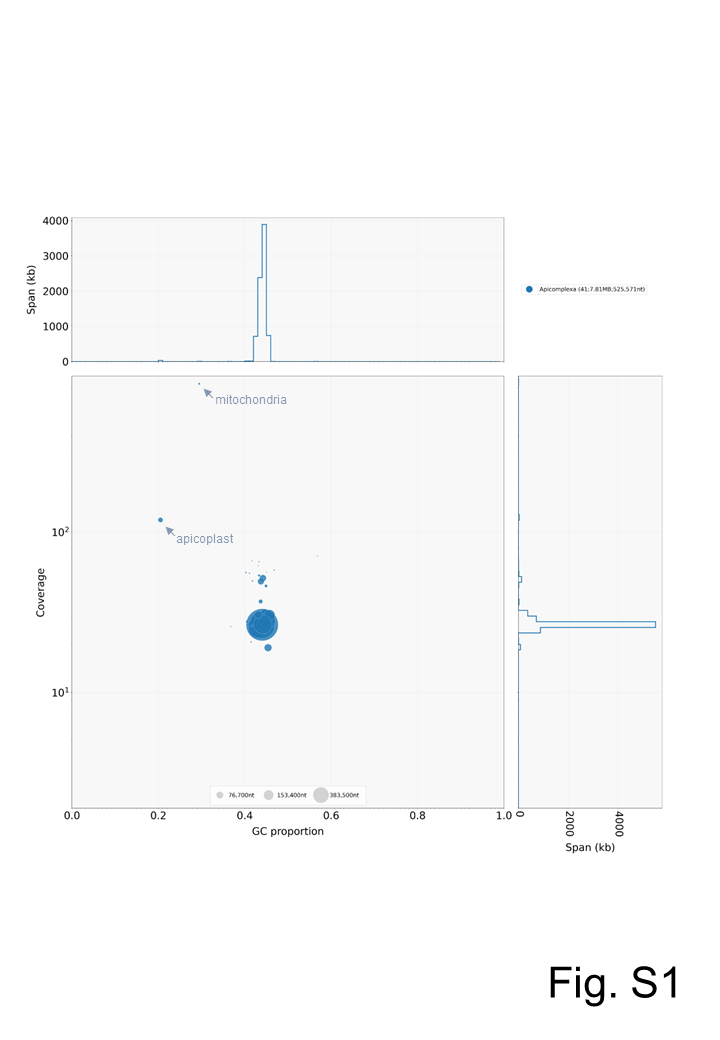

Supplement: Supplementary Figure 1 — Profile of the 41 assembled contigs of B. ovis by BlobTools. It was confirmed that no contigs were found to have low sequence depth or deviate from the average GC% except apicoplast and mitochondrial genome. [file Image_1.tif]

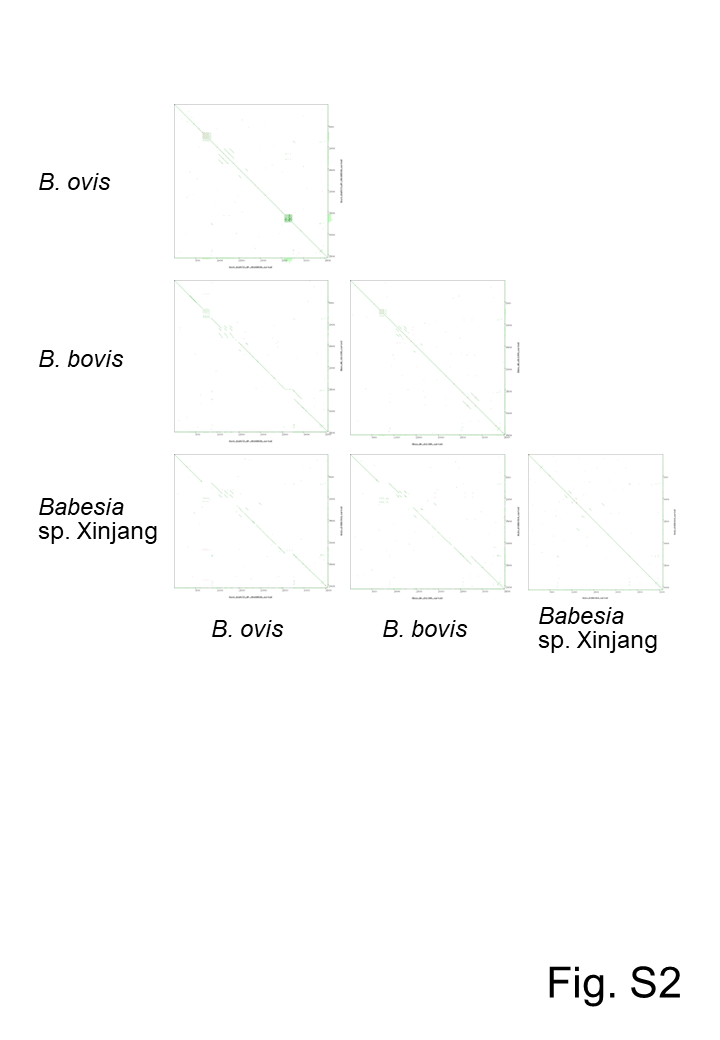

Supplement: Supplementary Figure 2 — Dotplot among apicoplast genomes of B. ovis. B. bovis and Babesia. sp. Xinjiang. [file Image_2.tif]

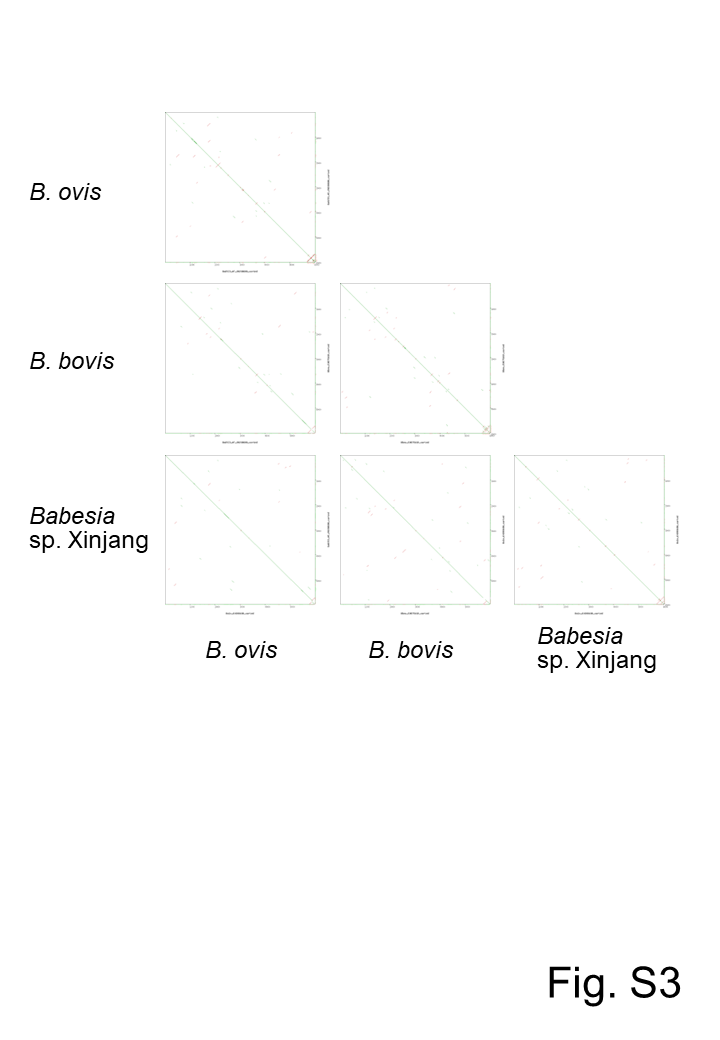

Supplement: Supplementary Figure 3 — Dotplot among mitochondrial genomes of B. ovis. B. bovis and Babesia. sp. Xinjiang. [file Image_3.tif]

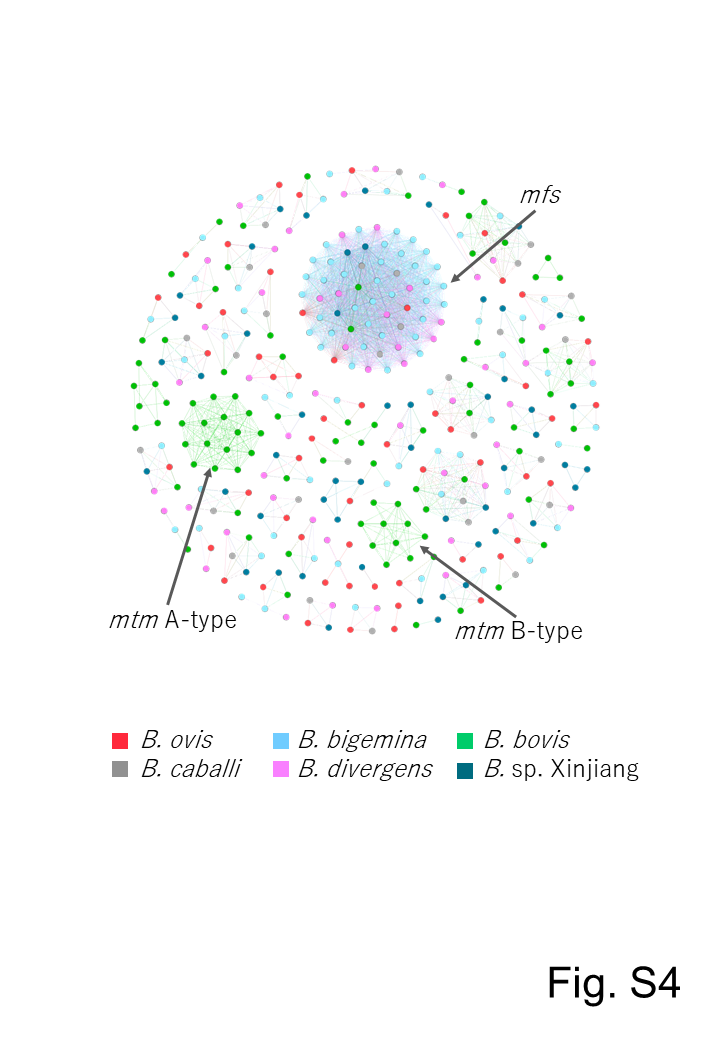

Supplement: Supplementary Figure 4 — Clustering based on sequence of genes with more than eight multi-transmembrane domains in B. bigemina, B. caballi, B. divergens, B. bovis, Babesia sp. Xinjiang, and B. ovis. Each node and edge represent a protein-coding gene and similarity between connected nodes, respectively. [file Image_4.tif]

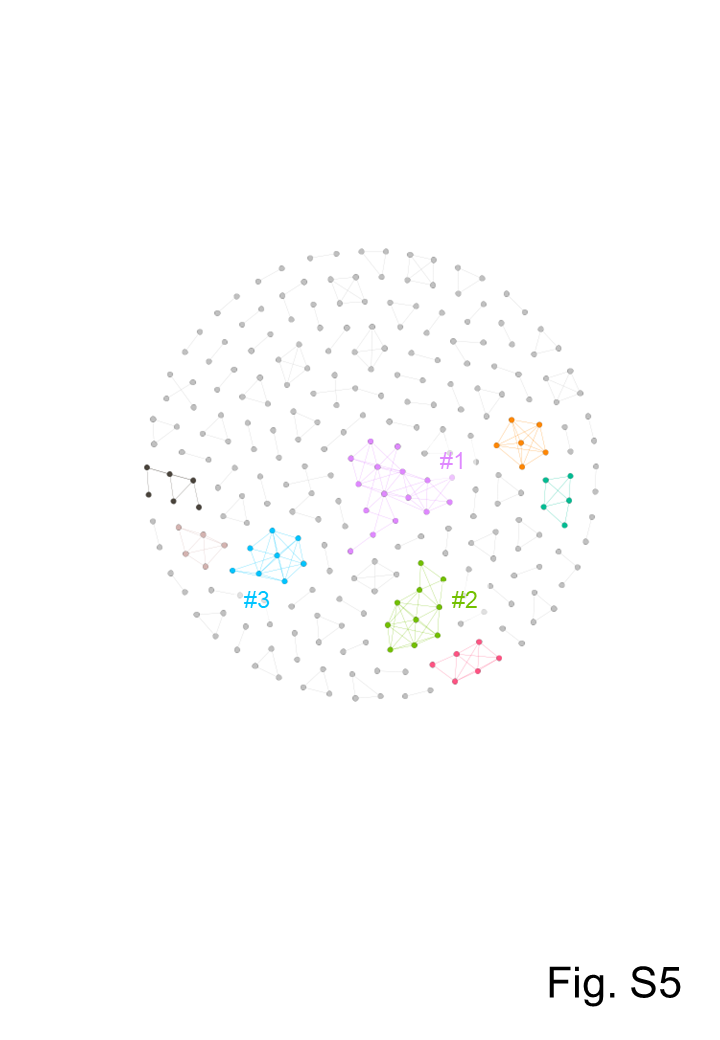

Supplement: Supplementary Figure 5 — Clustering based on sequence in B. ovis genes. Each node and edge represent a protein-coding gene and similarity between connected nodes, respectively. The clusters were colored if they consisted of five or more genes. [file Image_5.tif]
